# Large multimodal model‐based standardisation of pathology reports with confidence and its prognostic significance

**DOI:** 10.1002/2056-4538.70010

**Published:** 2024-11-15

**Authors:** Ethar Alzaid, Gabriele Pergola, Harriet Evans, David Snead, Fayyaz Minhas

**Affiliations:** ^1^ Department of Computer Science University of Warwick Coventry UK; ^2^ Histopathology Department University Hospitals Coventry and Warwickshire NHS Trust Coventry UK; ^3^ Warwick Medical School University of Warwick Coventry UK

**Keywords:** information extraction, pathology reports, report standardisation, large language models, LLM, large multimodal model, LMM, GPT‐4

## Abstract

Despite the existence of established standards and guidelines for pathology reporting, many pathology reports are still written in unstructured free text. Extracting information from these reports and formatting it according to a standard is crucial for consistent interpretation. Automated information extraction from unstructured pathology reports is a challenging task, as it requires accurately interpreting medical terminologies and context‐dependent details. In this work, we present a practical approach for automatically extracting information from unstructured pathology reports or scanned paper reports utilising a large multimodal model. This framework uses context‐aware prompting strategies to extract values of individual fields, such as grade, size, etc. from pathology reports. A unique feature of the proposed approach is that it assigns a confidence value indicating the correctness of the model's extraction for each field and generates a structured report in line with national pathology guidelines in human and machine‐readable formats. We have analysed the extraction performance in terms of accuracy and kappa scores, and the quality of the confidence scores assigned by the model. We have also evaluated the prognostic value of the extracted fields and feature embeddings of the raw text. Results showed that the model can accurately extract information with an accuracy and kappa score up to 0.99 and 0.98, respectively. Our results indicate that confidence scores are an effective indicator of the correctness of the extracted information achieving an area under the receiver operating characteristic curve up to 0.93 thus enabling automatic flagging of extraction errors. Our analysis further reveals that, as expected, information extracted from pathology reports is highly prognostically relevant. The framework demo is available at: https://labieb.dcs.warwick.ac.uk/. Information extracted from pathology reports of colorectal cancer cases in the cancer genome atlas using the proposed approach and its code are available at: https://github.com/EtharZaid/Labieb.

## Introduction

Information extraction and standardised formatting of pathology reports are essential steps towards consistent interpretation of pathological information. However, this task is difficult in the case of unstructured and legacy reports, which commonly include handwritten notes and often fail to adhere to the International Collaboration on Cancer Reporting guidelines [1]. The diversity and complexity of these reports make it challenging to apply automated methods for information extraction, as these methods must accurately interpret medical terminologies and context‐dependent details.

Early efforts to extract information from pathology reports applied conventional natural language processing techniques to extract useful information such as tumour site [2, 3, 4]. Such approaches tend to focus narrowly on extracting a single field or two out of all potential information contained in the report. Additionally, these models often struggle with generalisability where the performance can drop across datasets or domains due to variations in language, context, and terminology.

Large language models (LLMs) have shown promise in the domain of pathology, as they can capture pathological context and do not require additional labour‐intensive training for specific tasks. These models have been used in information extraction from pathology reports [5, 6, 7]. However, current LLM approaches often attempt to extract all fields simultaneously without providing sufficient context, which can result in inaccurate outputs. Furthermore, many of these approaches are limited to processing only text data as input, excluding image‐based reports. Additionally, they often do not adhere to standardised formatting when generating the output.

A significant challenge with LLMs is ensuring the reliability and correctness of the extracted information. Existing approaches for information extraction from pathology reports do not report confidence values of the fields they extract. Confidence estimation could serve as an indicator of whether the model is extracting the information correctly or not with higher confidence levels likely correlating with more accurate extractions. Another shortcoming of existing approaches is that it is not possible to use scanned reports as input thus limiting their applicability in use cases involving legacy systems.

**Figure 1:**
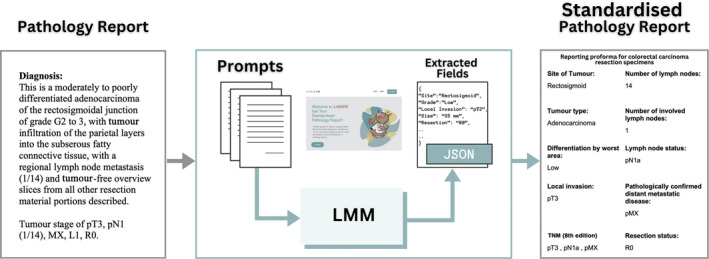
Standardisation of an unstructured text‐based pathology report with the proposed large multimodal model (LMM) framework (LABIEB). It takes as input pathology reports and a series of structured prompts to a LMM (GPT‐4 in this case). It then extracts specific fields from the responses and reformats the extracted information into a standardised format. The website is accessible at: https://labieb.dcs.warwick.ac.uk/.

Given these limitations, there is a need for a more comprehensive approach that can overcome these challenges. In this study, we propose a two‐stage information extraction and validation framework for extracting information from unstructured pathology reports using a large multimodal model (LMM). LMMs such as generative pretrained transformer‐4 (GPT‐4) [8] can offer a potential solution, as they can integrate and process both text and image data. The basic concept of this work is illustrated in Figure 1. We have used multiple prompts with adequate context to extract individual pieces of information, such as lymph node status and other specific fields. We expect this prompting technique to improve the overall performance of the model [9]. This architecture also enables the estimation of a confidence score to reflect the correctness of the extracted information for each field, allowing the user the liberty to reject extracted fields with lower confidence values. The output of the model follows established pathology reporting standards set by the UK Royal College of Pathologists (RCPath) [10] and can be saved in both human and machine‐readable formats. The proposed structure is tested on pathology reports for colorectal adenocarcinoma (COAD) patients from the cancer genome atlas (TCGA) dataset. In order to aid the reader in gaining a holistic understanding of this work, the major contributions of the paper are as follows:

1. A two‐stage architecture for information extraction and validation from unstructured pathology reports using LMMs.
2. Confidence assignment to extracted fields that is indicative of extraction correctness.
3. Analysis of prognostic value of structured reports and unstructured reports' embeddings.
4. Open availability for pathologists' use through a publicly available website.
5. Standardised reports for TCGA are made available for public use.

## Materials and methods

**Figure 2:**
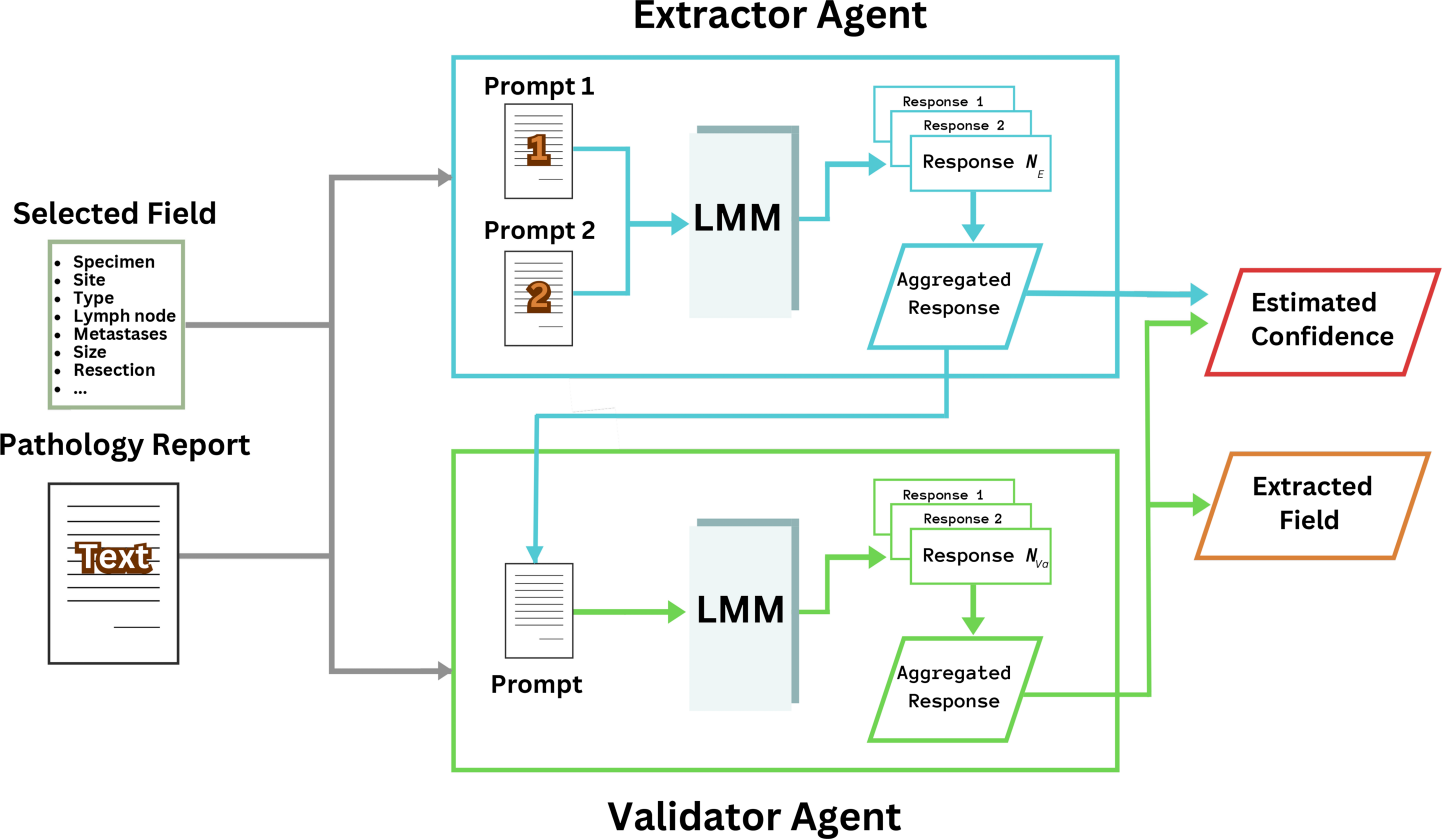
The framework for extracting information operates in two stages and takes an unstructured text report and a query field (e.g. grade or stage, etc.) as input and extracts the value of query field from the report along with assignment of a confidence value to the extracted field. First the Extractor operates by incorporating the input with two prompts to be sent to the LMM to produce $N_{E}$ responses which are aggregated into a single response. The same input along with the Extractor's response goes into the Validator's prompt and into the LMM. $N_{\mathrm{Va}}$ responses are generated and aggregated to a single response with a confidence value estimated by assessing both the Extractor's and the Validator's responses.

The architecture for the proposed model is shown in Figure 2. The methodology of the proposed model involves two‐stage prompting to two LMM agents we refer to as Extractor and Validator agents. The main function of the Extractor agent is to identify and extract the value of a specific field called the query field from the input text report. For example, we can extract grade, stage, or any other information directly from the report by specifying the query field to be grade or stage, respectively. The Validator's role is to assess and verify the accuracy of the Extractor's output. The final output of the model is a single response of the extracted field with an estimated confidence level that reflects the model's correctness and reliability in information extraction. Once various fields have been extracted, we can then analyse their prognostic significance.

### Dataset

We utilised the full‐text content of the pathologist reports for COAD cases from the TCGA dataset (https://www.cancer.gov/tcga) to perform experiments on the proposed model. It provides a multi‐centric collection of pathology reports with varying qualities and reporting styles. Out of N=635 COAD reports, 36 were filtered out due to their significant low image scanning quality. The total number of reports remaining in the experiment is $N_{T}=599$. The content of these unstructured text reports written by pathologists includes macroscopic description, microscopic description, as well as any diagnostic text fields available. We created a subset of $N_{V}=240$ validation cases where each field was manually extracted to assess the accuracy of the extractions from the proposed model.

### Reporting standards

Ensuring compliance with established standards for colorectal cancer reporting is essential for standardisation and maintaining consistency across all patients' data. We have followed the Standards and Dataset for histopathological reporting of colorectal cancer by the RCPath [11] to format the output. In addition, we have designed the prompts and aggregated the responses based on the categorisation schemes of the standards. We have extracted various fields in the standards such as specimen type, tumour type, tumour site, maximum diameter, local invasion status, histologic grade, number of examined lymph nodes, number of metastatic nodes, lymph node status, distant metastatic disease status, and resection status.

### Text extraction

The dataset consists of PDF reports scanned at varying image resolutions and with different textual lengths. Some reports include handwritten notes and others are in tabular format. For these files to be utilised by LMMs, they must first be transformed into text using optical character recognition (OCR). Conventional OCRs, such as the widely recognised Tesseract library [12], encountered difficulties in processing the diverse content effectively. We used the GPT‐4 Vision model to address this issue as it is recognised for its strong performance with Latin‐based OCR [9].

### Extractor agent

The Extractor is a GPT‐4‐Turbo powered agent aiming to extract a single field at a time from the textual report as illustrated in Figure 2. For each query field q, this agent sends two prompts to the LMM model to retrieve $N_{E}=20$ responses in total in JavaScript object notation (JSON) format. The agent then returns a single response that appeared the most among all responses. The Extractor confidence $E_{\text{Confidence}}$ is reported as the percentage of times the returned response appeared among responses for the query field. The model is instructed to respond with ‘Not Available’ when there is no sufficient information in the report. It is possible for the model to encounter ties since it returns the query field based on how many times it appeared, the interested reader can refer to supplementary material, File S1 for details on how the model resolves ties.

### Validator agent

The Validator is a GPT‐4‐Turbo powered agent used to validate the Extractor's response. This agent receives a prompt with instructions to validate the Extractor's response. For every query field, the Validator produces an output consisting of three labels: Correctness, Confidence, and Corrected. Correctness states whether the Extractor is correct, Confidence shows how confident the model is in its response (out of 100), and Correction is the Validator's response as a correction in case the Extractor was incorrect. The LMM produces $N_{\mathrm{Va}}=10$ responses in total in JSON format for a single prompt and for each query field. Responses are aggregated by the Validator and ties are handled with the same manner as the Extractor agent. The Validator confidence is reported as the percentage of times the returned response appeared among responses for each output label and each query field.

### Prompt design

**Figure 3:**
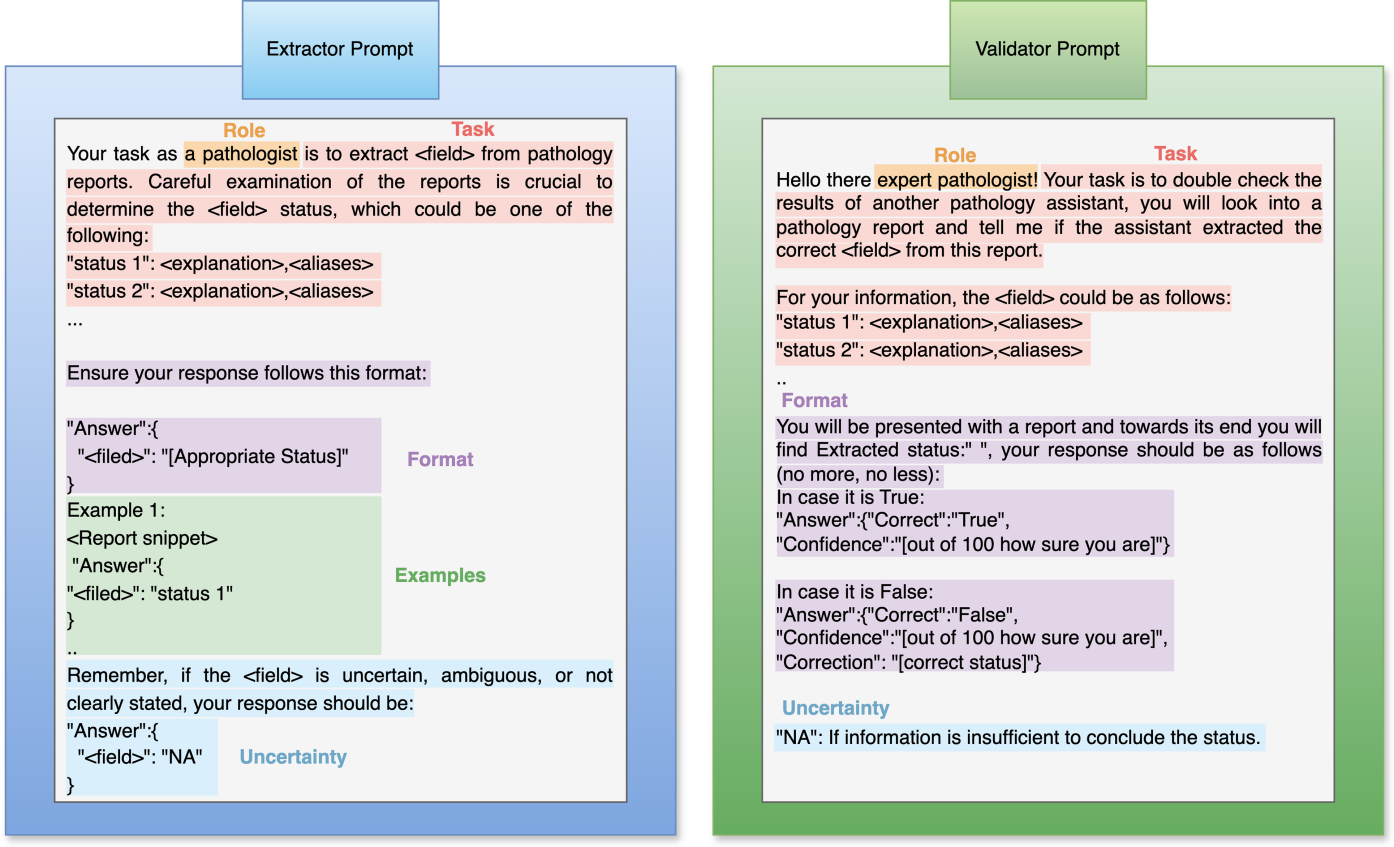
Basic structures of the Extractor and the Validator agents' prompts. Created with draw.io.

The prompt for information extraction is composed of five main components: role, task, format constraints, examples, and uncertainty handling. Two variations of designs are shown in Figure 3 with samples of both the information extraction and validation prompts. For each query field, we have designed two prompts for the Extractor and one for the Validator to utilise LMM's sensitivity to prompt format in confidence estimation [10]. Sample prompts used in the model are provided in the supplementary material, File S1 and in the online code repository: https://github.com/EtharZaid/Labieb.

### Role and task specifications

The role of the agents is specified as pathologists with the task of extracting or validating a specific field from a pathology report. We explain the task in detail reflecting the RCPath guidelines in the categorisation of each field. Each category is described along with its variations or aliases that the model might encounter while analysing the text for field extraction. For instance, the stage ‘pT4a’ is explained as ‘Tumour cells breaching the serosa or perforating’ and may also be referred to as ‘T4A’, ‘pT4’, or ‘T4’.

### Response format

We have defined the model's response format and instructed the model to follow this format when it generates the response. The specified response format is set to JSON where the query field is stated and followed by the extracted field. Specifying the format reduces output formatting errors, this reduction can be attributed to the fact that outlining the response structure limits the model's undesired creativity and directs its efforts on the specified task [13].

### Example scenarios

We have provided the model with a set of example scenarios for each query field where each example includes a segment of a report and the response we expect the model to generate. For instance, in the local invasion prompt, we have given a segment of a report stating (‘Tumour invades muscularis propria’) followed by the expected response (‘Local Invasion’: ‘pT2’). This shows the model that when a similar report is encountered, the model should generate a similar response. Including examples is referred to as in‐context learning, and it can enhance responses by guiding the model to adhere to the specifications of the expected response [14].

### Uncertainty handling

In the last component of the prompt, we have specified for the model how to handle ambiguous reports, confusing cases and reports with insufficient information. We have mentioned in the prompt that such cases exist, and the model could respond with ‘Not Available’ if it encountered one. It is crucial to provide the model the option to ‘not provide an answer’ or it might forcibly generate an answer, potentially leading to incorrect extraction.

### Confidence estimation and rescaling

Estimation of confidence is crucial in reflecting how reliable a response is. While having a model that generates accurate output is important, it is also important that such model is able to reflect its reliability as a measurement of confidence. The confidence of the final response made by the Validator for each query field q is estimated upon multiple factors as shown in Equation (1).

(1) $$C_{q}=\begin{array}{l} \frac{E_{\text{Confidence}}+V_{\text{Correct}}+V_{\text{Confidence}}+V_{\text{Correction}}+V_{\%\text{Correct}}}{5} \end{array}\;\times 100$$

Here, $E_{\text{Confidence}}$ corresponds to the confidence reported by the Extractor, $V_{\text{Correct}}$, $V_{\text{Confidence}}$, and $V_{\text{Correction}}$ are the confidences reported by each label in the Validator's response. In the equation, the term $V_{\%\text{Correct}}$ represents the frequency where the Validator's response confirms the Extractor's output is correct, reflecting a greater confidence in the response when there is agreement between both agents. The final value of $C_{q}$ ranges from 0 to 100, with 0 indicating the highest probability of extraction error and 100 indicating the highest probability of correct extraction. Relying on one factor only may not provide an accurate representation of the model's reliability as LMMs often exhibit overconfidence [15]. Furthermore, consistency in responses from a single prompt does not necessarily guarantee reliability, as these models can be consistent while being incorrect. We have averaged all factors as we expected the model to reflect its incorrectness on one or more of the factors due to the distinct ways of prompting (extraction and validation).

Estimated confidence is expected to be high as LMMs tend to be overconfident and consistent. Therefore, we have applied Platt's probability calibration to rescale confidence values of each query field $C_{q}$ and make it more representative of the actual performance of the model [16]. Platt's scaling works by fitting two coefficients $\left(A_{q},B_{q}\right)$ to the sigmoid function and use it to generate scaled confidence values to be reflective of the errors made by the model.

### Quantification of extraction accuracy

We have evaluated the model's performance by measuring both accuracy and Cohen's kappa statistics [17]. For each extracted value from a given query field in a report in the validation set, we assigned a label of 1 or 0 for correct and incorrect extractions, respectively. The mean value of these labels is the accuracy of the model. To calculate the kappa score, we have measured the agreement between the model extraction and the manual extraction of the same field in the validation set. The result is a score ranging from −1 to 1, where positive values indicate good agreement between the two and negative values means chance agreement. Kappa scores offer a more precise measure as it accounts for the possibility of agreement occurring by chance.

### Measurement of extraction performance and effectiveness of confidence

It is expected for LMMs to make mistakes considering the complexity and the extensive context needed to perform extraction tasks. We have evaluated the model's effectiveness by examining how accurately its confidence estimates reflect its correctness. We used the model's confidence scores along with the validation labels (0 or 1) to compute and plot the area under the receiver operating characteristic (AUROC) curve for each field [18]. This measure provides an assessment of the confidence assigned by the model as an indicator of the model's correctness in extractions. In addition, we hypothesised that excluding extractions with low confidence values would enhance the accuracy of the model. To test this hypothesis, we calculate the percentage of extractions *rejected* for falling below a specified confidence threshold and assess the accuracy of the model over *accepted* extractions that meet or exceed this threshold. The mean performance of the model is expected to increase with increasing confidence value thresholds resulting in the model effectively abstaining from producing a response when the generated response is expected to be incorrect.

### Analysis of prognostic value of standardised reports

The information contained in pathology reports is crucial for medical decision‐making and holds prognostic value [11]. In order to test this hypothesis, we have performed survival analysis to confirm the prognostic value of the standardised reports. For this purpose, we used a transductive survival ranking model [19] to stratify patients into high‐ and low‐risk groups based on disease‐specific survival endpoint. To evaluate the results, concordance index (*c*‐index) [20] was calculated by comparing the scores of the survival ranking model over all pairs of patients relative to their actual survival times. A *c*‐index of 1 implies perfect concordance between model predicted and actual survival times for patients whereas a concordance index of 0.5 corresponds to a random prognostic baseline. We have also plotted Kaplan–Meier (KM) survival curves [21] to visualise the capability of the standardised reports to stratify patients effectively into two distinct risk categories.

## Results

In this section, we present both qualitative and quantitative results from the proposed approach in terms of its ability to extract relevant information from pathology reports, the quality of its extractions, effectiveness of its confidence estimation for different fields as well as the prognostic significance of reports through survival analysis.

### Extraction of information from pathology reports

**Figure 4:**
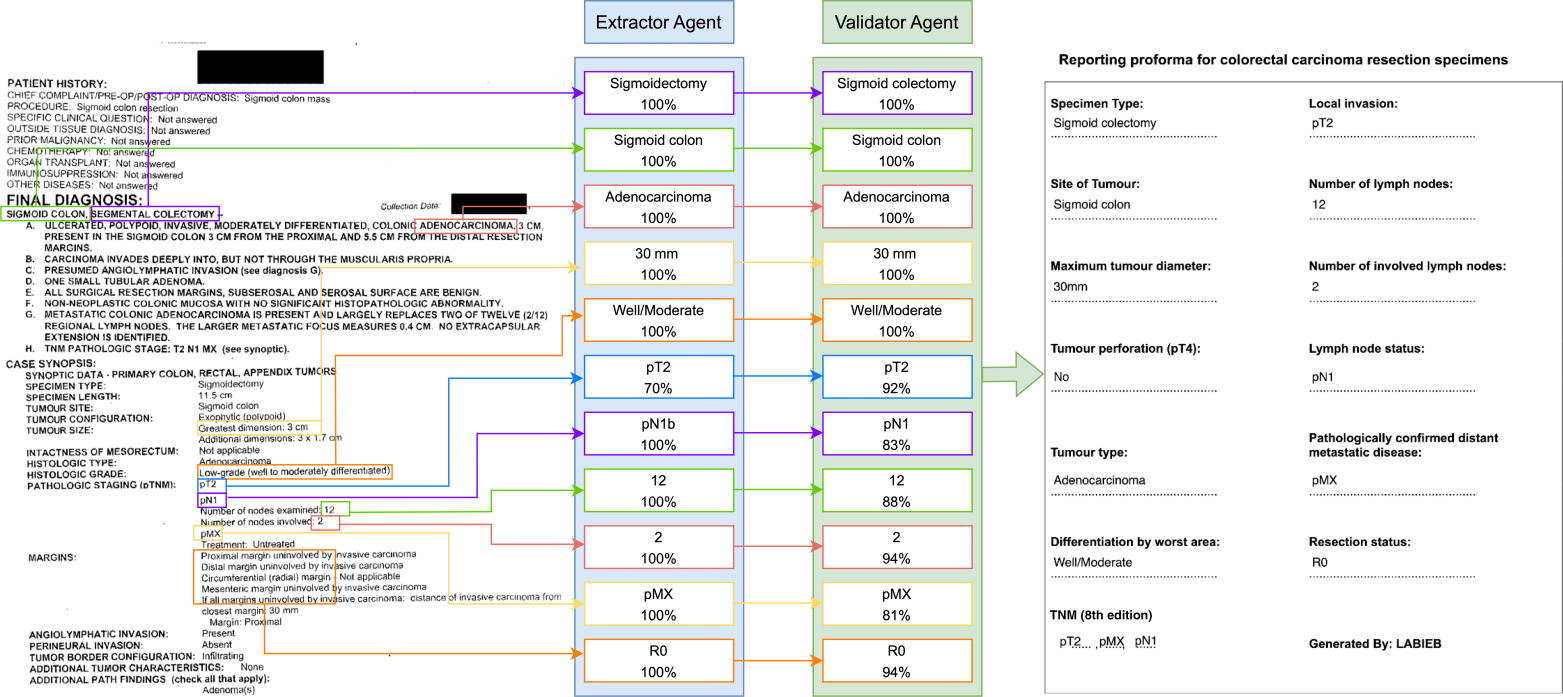
Sample of a colorectal adenocarcinoma report and how the model produces the output. The fields are extracted from the report by the Extractor agent and passed to the Validator which returns each field with its corresponding confidence value. The final output of the model is formatted according to the standards of reporting. Created with draw.io.

Figure 4 shows a sample report from the TCGA‐COAD dataset and the information extracted by the proposed approach. Each field is extracted with its corresponding confidence score and the final output of the model is shown following RCPath reporting proforma document. In addition, the model generates both text and JSON output to facilitate both human and machine readability or interpretation. More samples can be found in the supplementary material, File S1, which shows that the model can be an effective tool in automatic information extraction as well as standardisation of pathology reports. Extracted information with corresponding confidence estimates can be found in the supplementary material, File S2.

### Quantification of extraction accuracy

We measured the level of agreement between the model extractions and the manual extractions using both kappa statistics and accuracy; the scores are reported in Table 1. All kappa scores are positive (>0.5) and accuracies above 0.81 in most fields. Highest scores are for the ‘histologic grade’ field, where accuracy and kappa scores were 0.99 and 0.98, respectively, indicating little to no disagreement between the model's extraction and the actual values. This high percentage of correct extractions for this field in particular is attributed to its clear definition as the response should either be 0 (low grade) or 1 (high grade), which lowers the chances of errors.

**Table 1:** Results showing the accuracy and kappa scores of extracting different fields from pathology reports using the large multimodal model pipeline used in this work in comparison to the manually extracted value of the field for a validation set of *n* = 240 reports

Field	Set of possible values for the field	Kappa score	Accuracy
Histologic grade	High, low, NA (if not available)	0.98	0.99
Examined nodes	Numerical (0–116), NA (if not available)	0.92	0.92
Metastatic nodes	Numerical (0–50), NA (if not available)	0.91	0.92
Tumour site	Caecum, ascending colon, hepatic flexure (or right flexure), transverse colon, splenic flexure (or left flexure), descending colon, sigmoid colon, rectosigmoid, rectum, NA (if not available)	0.87	0.89
Maximum diameter	Numerical (0–160), NA (if not available)	0.85	0.87
Tumour type	Adenocarcinoma, mucinous adenocarcinoma, NA (if not available)	0.74	0.94
Local invasion status	pTIS, pT0, pT1, pT2, pT3, pT4, pTX (if not available)	0.86	0.94
	pTIS, pT0, pT1, pT2, pT3, pT4a, pT4b, pTX (if not available)	0.67	0.84
Lymph node status	pN0, pN1, pN2, pNX (if not available)	0.91	0.92
	pN0, pN1a, pN1b, pN1c, pN2a, pN2b, pNX (if not available)	0.55	0.61
Distant metastatic disease	pM0, pM1, pMX (if not available)	0.86	0.94
	pM0, pM1a, pM1b, pM1c, pMX (if not available)	0.54	0.85
Specimen type	Total colectomy, subtotal colectomy, right hemicolectomy, transverse colectomy, left hemicolectomy, sigmoid colectomy, Hartmann's procedure, anterior resection, abdominoperineal excision, other	0.54	0.61
Resection	R0, R1, R2, RX (if not available)	0.50	0.81

The table shows the sets of unique values possible for each field. For fields like local invasion status, lymph node status, and distant metastatic disease, performance was analysed at different levels of granularity in terms of broad and more specific categories (e.g. pT4a and pT4b versus only pT4 in local invasion status).

We observed high variability in the reporting of the ‘specimen type’ in the reports, as some of the reports were not originally written in English and a number of terms were lost in translation. This has led to lower scores for this query field. While having a considerably high accuracy of 0.81, the ‘Resection’ field has the lowest kappa score. This was a result of the model incorrectly assuming a resection status of ‘R0’ when no information about a status was in the report. However, upon closer inspection, the estimated confidence for these incorrect extractions was found to be low, indicating that the model has correctly identified its inability to correctly extract such information. For fields like lymph node status, local invasion, and metastatic disease, we analysed the model's performance at various levels of granularity, including broad and specific sub‐categories with the model showing higher accuracies in extracting broad category values for these fields.

### Quantification of confidence assignment performance

**Figure 5:**
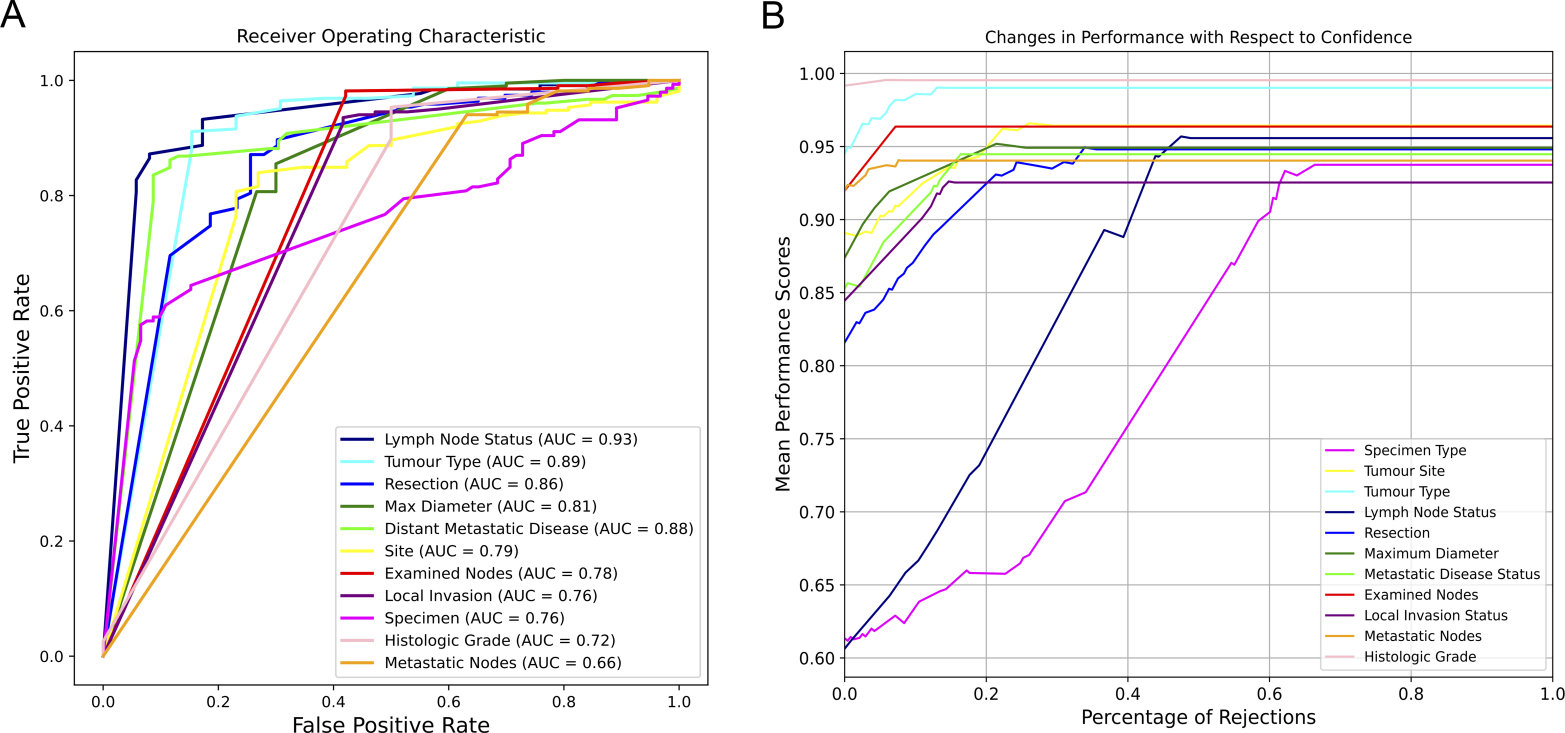
(A) Receiver operating characteristic curves for extracted fields plotted to measure how reflective the estimated confidence of the model's performance. AUROC curve value for each field is shown in the legend. (B) Plot of mean performance when samples are rejected based on their confidence.

In order to assess if the confidence values estimated by the model for different fields allow us to identify potential extraction errors, we calculated the AUROC curves presented in Table 2 and plotted the corresponding curves in Figure 5A. A high value of the AUROC curve implies that the confidence assigned by the model for a given field is high if the field has been correctly extracted by the model. The highest AUC of 0.93 was observed for the lymph node status field, which showed a low accuracy of 0.61. For this field, the model made a larger number of extraction errors but due to the confidence assignment by the model such errors can be detected. On the other hand, while the model correctly extracted histologic grade with a high accuracy of 0.99, the assigned confidence was less indicative of correctness compared to other fields, resulting in lower AUROC curve values. Similarly, metastatic nodes had the lowest AUROC curve value of 0.66, despite the model performing at a high accuracy of 0.92. This shows a challenge in providing a reliable confidence estimate when accuracy is high.

**Table 2:** AUROC curve scores for all extracted fields

Field	AUROC	Field	AUROC	Field	AUROC
Lymph node status	0.93	Distant metastatic disease	0.81	Specimen type	0.76
Tumour type	0.89	Site	0.79	Histologic grade	0.72
Resection	0.86	Examined nodes	0.78	Metastatic nodes	0.66
Maximum diameter	0.81	Local invasion status	0.76		

The scores evaluate whether the confidence value assigned by the model indicates the model's ability to correctly identify when an extraction error has been made. Higher AUROC curve means a good confidence estimate that is indicative of the correctness of the extraction.

### Abstention analysis curves

We analysed how the accuracy of extraction changes as those fields with low confidence values are rejected to confirm that the confidence values produced by the model can be used to reject fields that are not extracted correctly. The percentage of *rejected* samples are plotted against mean performance score in Figure 5B. The plots clearly demonstrate a positive trend indicating that as the rejection rate increases, the model's performance improves accordingly. This suggests that selectively considering extractions with higher confidence scores can significantly improve overall performance. The trend is clearer with lymph node status due to its high AUROC curve value discussed previously and the confidence being more indicative of errors. It is less apparent in histologic grade due to the effect of the model's low extraction errors.

### Prognostic value of pathology reports

**Figure 6:**
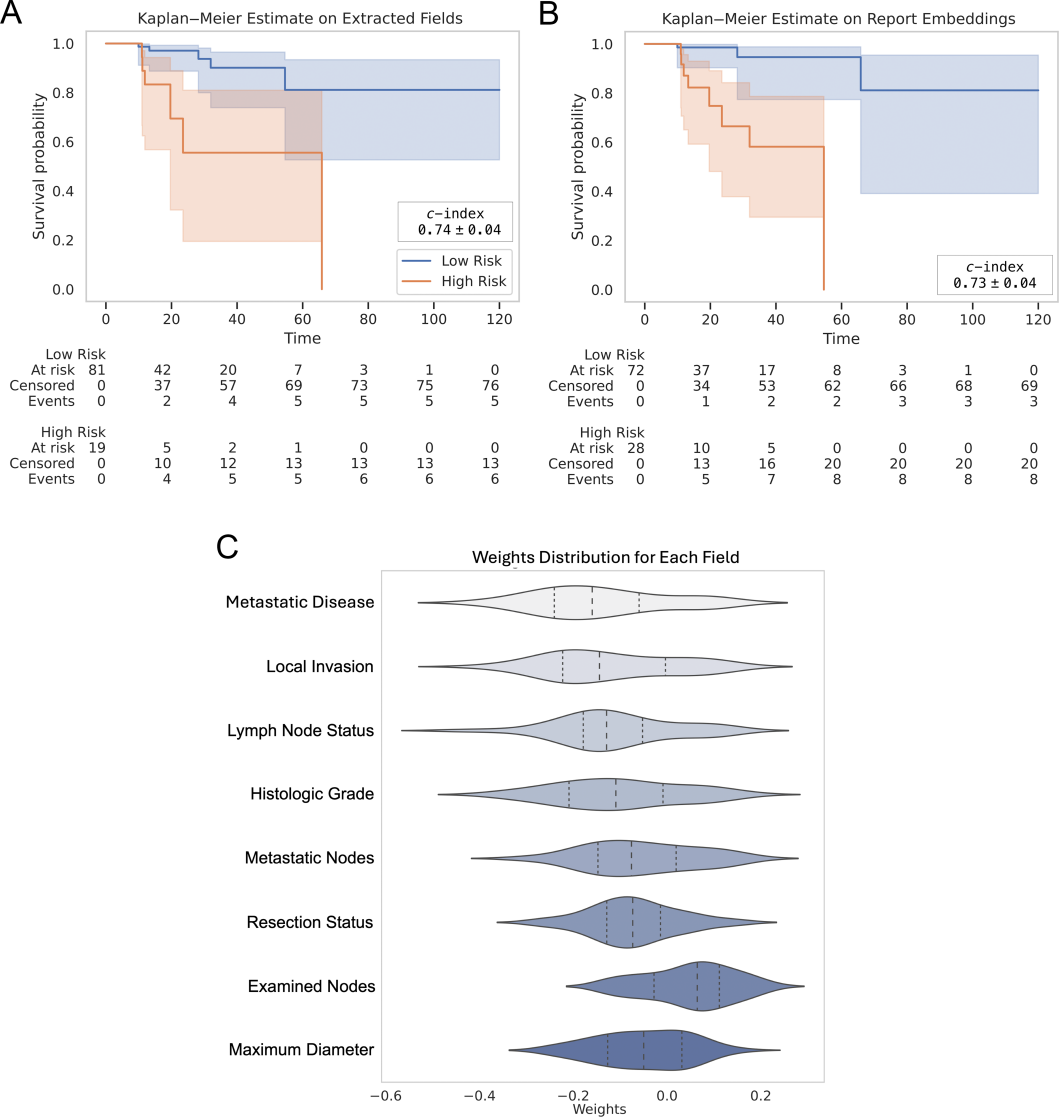
(A and B) Kaplan–Meier survival curves comparing high‐risk and low‐risk groups over time for survival probabilities based on (A) extracted fields for standardised reports and (B) report embeddings. (C) Violin plot for the distribution of weights of the standardised report fields for the survival model.

We analysed the prognostic value of different fields in pathology reports. Based on the automatically extracted fields by the proposed approach, we have been able to stratify patients into two low‐ and high‐risk groups automatically as shown in the KM curves plotted in Figure 6A. It can be noted that the survival time of the low‐risk group is significantly higher than the high‐risk group with a *p* value of ≪0.005. This survival prediction based on extracted field values results in a concordance index of 0.74 ± 0.04. This high *c*‐index indicates that the content of the reports is a reliable predictor of survival with strong prognostic relevance in assessing patient outcomes, which is essential in planning proper intervention or treatment.

We also found that feature embeddings of unstructured or raw‐text pathology reports obtained by an LMM (OpenAI ‘text‐embedding‐3‐small’) are highly prognostic. The stratification based on these embeddings into high‐ and low‐risk patients is shown in Figure 6B and is statistically significant with p≪0.002 and a comparable *c*‐index of 0.73 ± 0.04.

In addition, we performed univariate analysis on each field in the standardised report to measure their contribution to the patient survival outcome prediction. Figure 6C visualises the weight distributions for each field. Fields are ranked from top to bottom with the top having the highest weight values, thus contributing the most to the survival score generated by the survival model. The central vertical line in each field represents the median of the weight distribution. The top three fields are metastatic disease, local invasion, and lymph node status, which align with the TNM staging system known to describe the progression of cancer. TNM is used to determine the best treatment plan for the patient [22]. This alignment affirms the standardised reports' value and its clinical relevance in accurately predicting patient outcomes. A more detailed plot for the weights can be found in the supplementary material, File S1.

## Discussion

In this work, we have proposed a two‐stage information extraction and validation from unstructured pathology reports using LMM. We demonstrated the consistent and reliable performance of the model through high accuracy and kappa scores. The results show that this framework is effective in estimating a confidence score that is representative of the correctness of the extracted information. For each extracted field, the assigned confidence gives the user of the extracted data the liberty to reject fields with lower confidence. An interesting finding from this work is that the extracted fields are predictive of survival. This suggests the extracted information can capture key prognostic elements that are significant in patient outcomes. Another interesting finding was that the report text embeddings are also prognostic. We have made the framework available on a user‐friendly website, enabling pathologists to easily structure and standardise their reports with an option to handle other cancer types in the future.

Future research in this domain can focus on developing methods to automatically convert pathology reports between different standards, enhancing interoperability across healthcare systems and ensuring consistency in data reporting and analysis. Additionally, gaining access to large‐scale repositories of pathology reports can provide a robust foundation for fine‐tuning LMMs, potentially improving accuracy and allowing for better validation across various report types and patient populations. Furthermore, the development of interactive agents to assist in report writing represents another interesting future direction. These agents could be designed to provide real‐time feedback and automated analysis during the documentation process.

In conclusion, the proposed framework demonstrates promising potential and is versatile enough to be employed in numerous information extraction tasks from unstructured text. Structuring and standardising reports is expected to enhance the sharing of diagnostic and treatment information among medical professionals.

## Author contributions statement

EA and FM conceptualised the study. EA conducted and evaluated the experiments with the guidance of FM. GP provided feedback and advice for the experimental set‐up. HE and DS assisted in the validation of extracted information. All authors corrected and agreed to the final version of the manuscript.

## Supporting information


**File S1.** Additional experimental details

**File S2.** Extracted information with confidence estimations

## Data Availability

The dataset TCGA‐COAD is available on TCGA Program under https://www.cancer.gov/ccg/research/genome-sequencing/tcg. The code for using the GPT‐Turbo application programming interface with a prompt and performing downstream analysis is available on our GitHub repository at the following URL: https://github.com/EtharZaid/Labieb.
