# Supplementary material for: Large multimodal model‐based standardisation of pathology reports with confidence and its prognostic significance
Source: J Pathol Clin Res. 2024 Nov 15;10(6):e70010. doi: 10.1002/2056-4538.70010 (PMC11565444; doi:10.1002/2056-4538.70010)
Supplement: Supplementary file 1 — File S1. Additional experimental details [file CJP2-10-e70010-s001.pdf]

Large multimodal model-based standardisation of pathology reports with confidence and its prognostic significance

E Alzaid *et al*. *J Pathol Clin Res* https://doi.org/10.1002/2056-4538.70010

Supplementary File S1

Additional Experimental Details

Ties Handling in Responses

The model may encounter ties since it returns the query field based on how many times it appeared. A specific protocol based on the query field is followed to resolve such ties. In case the query field was either Lymph node status or distant metastatic disease status, tied responses that have an identical numerical designation but belong to different subcategories, the response with the broader range is chosen (for example, pN1 is preferred over pN1a). This would help minimise the chances of reporting the incorrect response by cautiously reporting the response that covers the others (for example, pN1 covers pN1a). For ties in query fields with numerical values (such as maximum diameter or number of examined nodes), the selected response is the response with the largest value (for example, select maximum diameter of 45mm over 30mm). For all query fields, if a tie occurs between a specific extracted field and a response indicating that the information is "Not Available", the extracted field's data is given precedence.

Samples of Standardised Reports

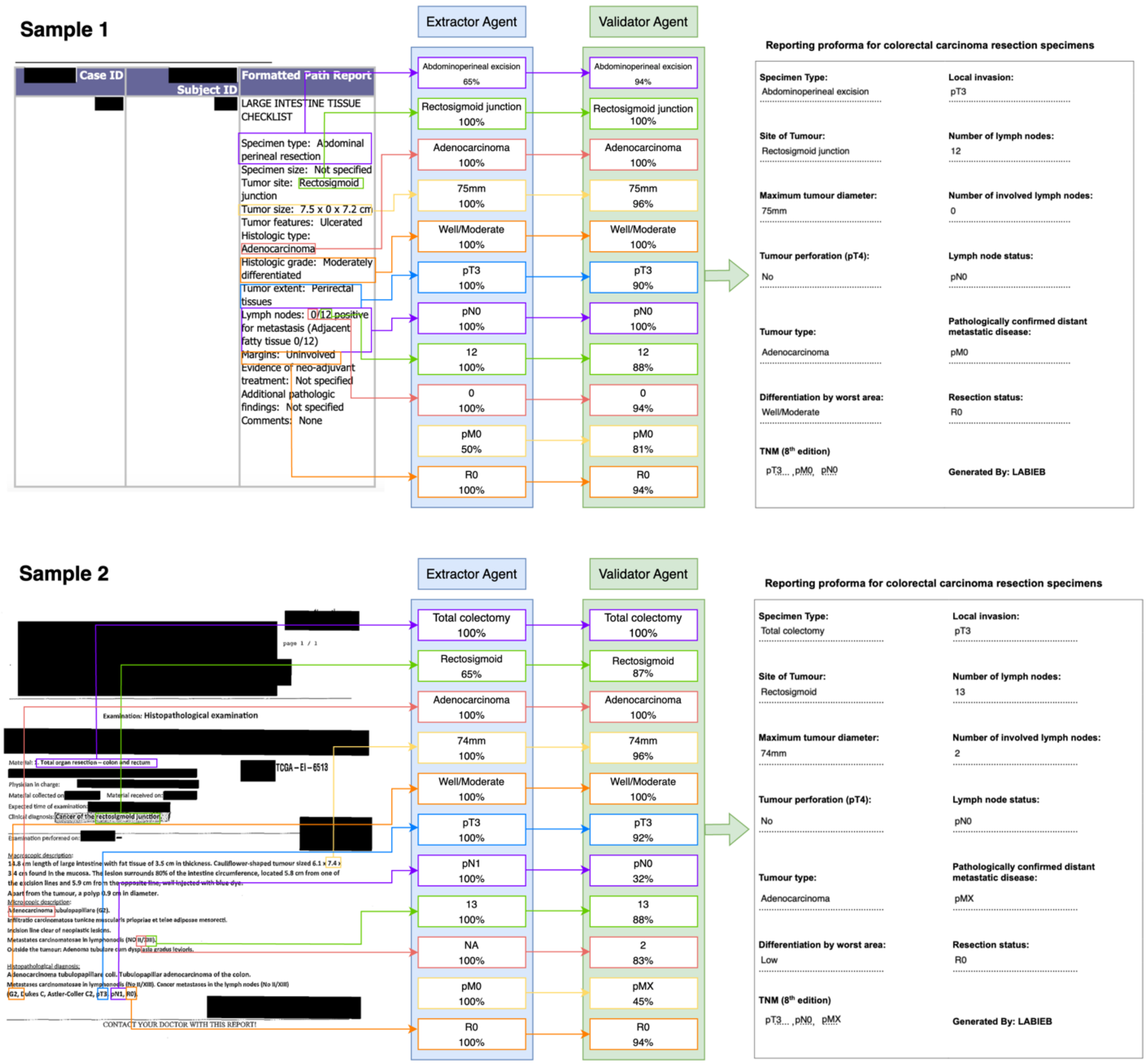

More samples of the model's standardisation process. Note the varying reporting structure where sample 1 is in a tabular format and sample 2 is in free text. The LMM was able to accurately extract the text from both cases. The text in sample 2 is in both English and Italian and the model has shown capability of interpreting the report content by correctly extracting query fields. For incorrectly extracted fields (e.g. Lymph node status in sample 2) the estimated confidence is low which proves the significance of the confidence estimate as a performance indicator.

Field Value Importance in Prognosis

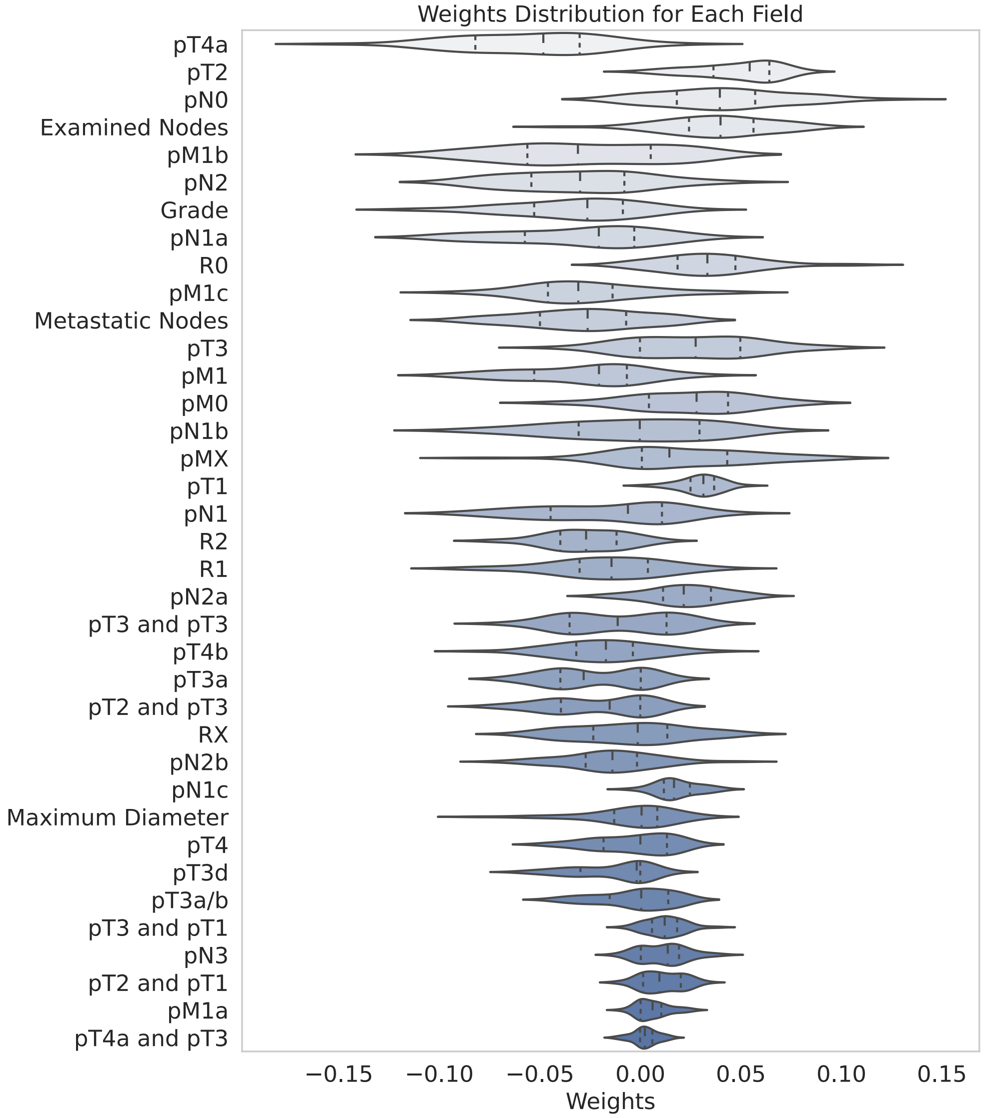

This figure shows the survival model weights of One-Hot Encoded extracted fields. The distribution of the weights across each field shows how relevant it is to the survival outcomes. The fields are sorted from top being the most relevant to the bottom being the least. The top field is an advanced tumour stage of “pT4a” with a negative weight, meaning lower survival rate for patient in this stage. On the other hand, the stage “pT2” and lymph node status of “pN0” have positive weights, meaning they are indicative of good prognosis.

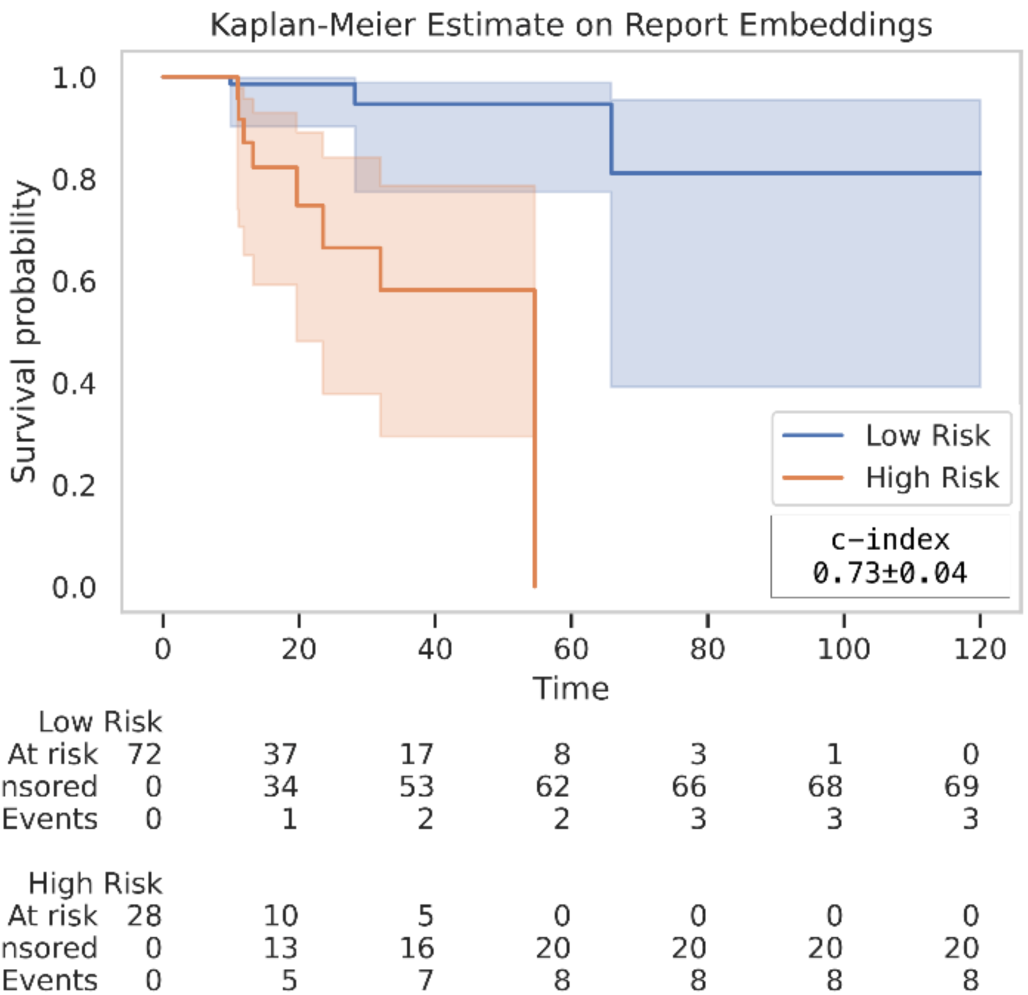

	0	20	40	60	80	100	120
**Low Risk**							
At risk	72	37	17	8	3	1	0
Censored	0	34	53	62	66	68	69
Events	0	1	2	2	3	3	3
**High Risk**							
At risk	28	10	5	0	0	0	0
Censored	0	13	16	20	20	20	20
Events	0	5	7	8	8	8	8

Analysis of Prognostic Value of Report Embeddings

We conducted another experiment to demonstrate the value of reports content by transforming the text into word embeddings which are the numerical representation of the text aiming to capture its meaning and context. Transforming text to this format is one way of handling unstructured text [1]. We have transformed text reports into 1536 dimensional embeddings using OpenAI "text-embedding-3-small" and performed survival analysis using the same method as the analysis for standardised reports. The c-index we have achieved is reasonably high for up to 0.73±0.04 for embeddings which is indicative of the actual prognostic value held within the report text. The KM curve is shown above, where the survival time of the low risk group is clearly higher than in the high risk group.

LABIEB Website

We have developed a web-page for our model to make this work accessible to everyone (https://labieb.dcs.warwick.ac.uk). We have chosen the name LABIEB (pronounced labeeb) which is an Arabic adjective synonym for the word "wise", taken from this proverb "A gesture for the wise suffices". We have based the name on this proverb as the proposed model is clever enough to perform the task with a fairly small set of instructions. The interface is intuitive and user-friendly to ensure accessibility for individuals across various levels of technical expertise. The page offers the user the option to upload the report file in either PDF or text format. The file will be processed through openAI GPT-4 Turbo API commands and the results will be displayed on the same page. The page provides multiple ways to download the results: JSON format, PDF format (follows the same template as the RCPath), and in text format.

Reference

1. Egger R. Text Representations and Word Embeddings. In Egger R, ed. Applied Data Science in Tourism: Interdisciplinary Approaches, Methodologies, and Applications. Cham: Springer International Publishing, 2022; 335-361.
